# Light at the end of the tunnel: Clinical features and therapeutic prospects of KRAS mutant subtypes in non-small-cell lung cancer

**DOI:** 10.3389/fgene.2022.890247

**Published:** 2022-10-21

**Authors:** Liyuan Gao, Weizhang Shen

**Affiliations:** Department of Oncology and Hematology, The Second Hospital of Jilin University, Changchun, China

**Keywords:** non-small-cell lung cancer, KRAS mutation, KRAS G12C, KRAS G12V, KRAS G12D

## Abstract

Lung cancer is one of the most common causes of cancer-related deaths, and non-small-cell lung cancer (NSCLC) accounts for approximately 85% of all lung cancer cases. Kirsten rat sarcoma virus (KRAS), one of the three subtypes of the RAS family, is the most common oncogene involved in human cancers and encodes the key signaling proteins in tumors. Oncogenic KRAS mutations are considered the initiating factors in 30% of NSCLC cases, accounting for the largest proportion of NSCLC cases associated with driver mutations. Because effective inhibition of the related functions of KRAS with traditional small-molecule inhibitors is difficult, the KRAS protein is called an “undruggable target.” However, in recent years, the discovery of a common mutation in the KRAS gene, glycine 12 mutated to cysteine (G12C), has led to the design and synthesis of covalent inhibitors that offer novel strategies for effective targeting of KRAS. In this review, we have summarized the structure, function, and signal transduction pathways of KRAS and discussed the available treatment strategies and potential treatment prospects of KRAS mutation subtypes (especially G12C, G12V, and G12D) in NSCLC, thus providing a reference for selecting KRAS mutation subtypes for the treatment of NSCLC.

## 1 General structure of the KRAS protein and the KRAS signaling pathway

**FIGURE 1:**
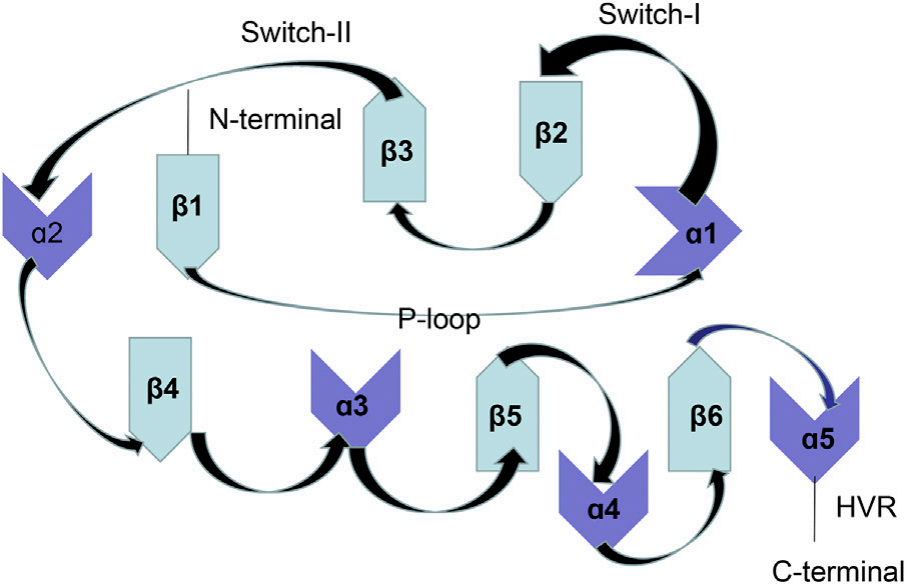
KRAS protein structure.

The activated RAS gene was first discovered in 1982, which is also the first identified proto-oncogene. The RAS gene family includes HRAS, KRAS, and NRAS, which are located on chromosomes 11, 12, and 1, respectively (Cox and Der 2010). KRAS is the most frequently mutated subtype. The RAS gene has three mutation sites, namely, G12, G13, and Q61. KRAS differs from NRAS and HRAS because it is the only RAS subtype that is predominantly mutated at 12 sites (Cox and Der 2010). KRAS encodes and manufactures the KRAS protein, which is a membrane GTPase. Structurally, the KRAS protein mainly includes a G-domain and C-terminal structural elements [also known as a hypervariable region (HVR)]. The G-domain includes a switch region (switch I and II) and a P-loop and forms the basis for biological functioning of the GTPase protein. HVR mainly anchors RAS to membranes (Hancock et al., 1990; Welman et al., 2000; Abraham et al., 2010). The switch region forms the binding interface for effector proteins and the regulatory factors of RAS such as GTPase-activating proteins (GAPs) and guanine nucleotide exchange factors (GEFs). The structure of the KRAS protein is shown in Figure 1.

**FIGURE 2:**
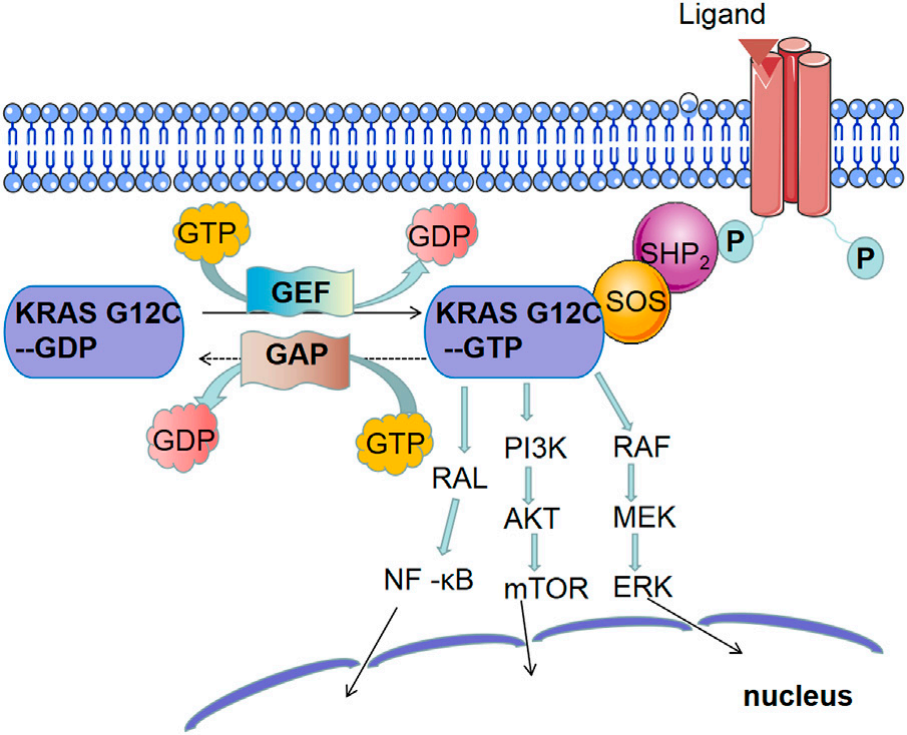
Main signaling pathway of KRAS mutations: ① MAPK signaling pathway: continuous activation of KRAS leads to the activation of RAF, the first kinase in the mitogen-activated protein kinase (MAPK) pathway, which phosphorylates MEK and then activates extracellular signal-regulated kinase (ERK). ERK both activates the cytoplasmic matrix and is transported to the nucleus to stimulate the expression of multiple genes involved in cell proliferation, survival, differentiation, and cell cycle regulation. Extensive studies have shown that MAPK signaling pathway plays an important role in RAS-mediated tumorigenesis. ② PI3K signaling pathway: phosphatidylinositol-4, 5-diphosphate trikinase (PI3K) also plays a key role in RAS-mediated tumorigenesis. When KRAS is activated, PI3K converts phosphatidylinositol 4, 5-phosphate (PIP2) to phosphatidylinositol 3, 4, 5-triphosphate (PIP3) through phosphorylation, which activates phosphoinositol-dependent kinase 1 (PDK1) and phosphorylates AKT, a serine/threonine-specific protein kinase. AKT activates mammalian target of rapamycin (mTOR). ③ NF-κB signaling pathway: At rest, NF-κB forms a complex with its inhibitory factors, which anchors NF-κB to the cytosol. When KRAS is activated, the RAL-NF-κB pathway is activated.

The KRAS protein can transmit extracellular signals to the nucleus and participate in the regulation of signaling pathways related to the growth, proliferation, differentiation, and apoptosis of tumor cells. Its activity is mainly mediated by guanosine triphosphate (GTP) or guanosine diphosphate (GDP) and is activated when bound to GTP and inactivated when bound to GDP. Because the GTPase activity of KRAS is not strong, GAPs are required to hydrolyze and inactivate KRAS-bound GTP (Vetter and Wittinghofer 2001). GAPs have an SH2 domain and can directly bind to the activated receptor. GDP release requires the participation of GEFs. Son of Sevenless (SOS1) is a GEF that promotes KRAS activation (Jeng et al., 2012). SHP2 is a protein tyrosine phosphatase that interacts with RTK and SOS1 to mediate KRAS activation (Mainardi et al., 2018). KRAS mutations promote the interaction between GAPs and KRAS, which promotes the gradual accumulation of active KRAS by inhibiting the hydrolysis of GTP, thereby breaking the regulatory cycle of active and inactive KRAS (Scheffzek et al., 1997). In addition, KRAS mutations can persistently activate the common KRAS-dependent downstream pathways, such as RAF/MEK/ERK, PI3K/AKT/mTOR, and RAL/NF-κB signaling pathways. The continuous activation of downstream molecules leads to tumorigenesis. The signal transduction pathway of the KRAS protein is shown in Figure 2.

Therapeutic targeting of KRAS mutations is difficult because of the structure and regulatory pathways of the KRAS protein (Canon et al., 2019; Fell et al., 2020; Goebel et al., 2020): ① The protein surface is excessively smooth, and only one GTP binding site is available. ② The lack of favorable small-molecule-binding pockets makes it difficult for drug molecules to compete with GTP. The binding of GTP to KRAS is very strong, reaching the level of Pmol, and the concentration of GTP in cells is very high, thus making it difficult for small-molecule inhibitors directly targeting GTP-binding pockets to compete with it. ③ KRAS is mainly regulated in the cell membrane. Owing to the aforementioned reasons, it is difficult for drug developers to find binding pockets for the small-molecule drug candidates on the surface of KRAS. Therefore, although KRAS is the earliest identified oncogene, the development of small-molecule inhibitors of mutated KRAS subtypes is difficult. For a long time, the KRAS protein was described as an “unavailable target.” However, the discovery of the KRAS G12C (glycine 12 mutated to cysteine)-targeted inhibitors AMG510 and MRTX849 offers novel strategies for effective targeting of KRAS.

## 2 KRAS mutation in non-small-cell lung cancer

KRAS-mutated tumors are the most common subtype of cancer with potentially targeted molecules in non-small-cell lung cancer (NSCLC) (Jordan et al., 2017). KRAS mutations are associated with smoking history, adenocarcinoma, and female sex among patients with lung cancer (Shepherd et al., 2013). Moreover, the incidence of KRAS mutations is different between the East and the West. KRAS mutations are found in approximately 20%–25% of lung cancer cases in the Western countries (Dogan et al., 2012; Shepherd et al., 2013; El Osta et al., 2019) and 10%–15% of lung cancer cases in Asian countries (Dearden et al., 2013; Yoshizawa et al., 2013).

KRAS mutations have been frequently discovered as driver mutations that are mutually exclusive with other driver genes, such as EGFR/ALK/BRAF/ROS1 (Renaud et al., 2016; Adachi et al., 2020; West et al., 2022). KRAS is co-mutated with other genes in 53.5% of cases. TP53, STK11, MET (amplification), ERBB2 (amplification), KEAP1, ATM, SMARCA4, ROS1, and PIK3CG are among the co-mutated genes. KRAS/TP53 co-mutation is the most common, accounting for approximately 39.4% of all cases (Scheffler et al., 2019). Additionally, studies have shown that patients with lung adenocarcinoma with TP53 and KRAS co-mutations have a higher programmed cell death-ligand 1 (PD-L1) expression than patients with either TP53 or KRAS mutation, suggesting that this population may benefit from anti-programmed cell death protein 1 (PD-1) immunotherapy (Dong et al., 2017).

A majority of KRAS mutations affect codons 12, 13, or 61. Mutations at codon 12 are the most common, occurring in > 90% of KRAS mutation-driven cancer cases. G12V (glycine 12 mutated to valine) and G12C are the two most common allelic mutations, accounting for > 50% of all KRAS mutation-driven cancer cases (Herdeis et al., 2021).

Among the subtypes of KRAS mutations, KRAS G12C and G12V mutations are epidemiologically associated with smoking history, whereas G12D mutations are more common among individuals who do not smoke. KRAS mutations can be divided into two types: transmutation (purine nucleotide substitution for a pyrimidine or *vice versa*) or transfer mutation (purine to purine or pyrimidine to pyrimidine substitution). Transmutation is more common among individuals who smoke, whereas metastatic mutations are more common among individuals who do not smoke (Tomasini et al., 2016).

## 3 KRAS G12C mutation

The KRAS G12C mutant subtype has a cysteine residue (glycine position 12 is mutated to cysteine) and is the most common KRAS mutation in NSCLC (Scheffler et al., 2019).

### 3.1 KRAS G12C-targeted drugs

Owing to the special characteristics of KRAS G12C mutations, specific irreversible inhibitors of KRAS G12C have been developed. Recently, various inhibitors that target KRAS G12C have been gradually introduced in clinical practice. At present, adagrasib and sotorasib are FDA-approved drugs for the treatment of patients with lung cancer with KRAS G12C mutation. To achieve therapeutic efficacy, the main mechanism of action is the binding of these drugs with KRAS G12C, which locks the oncoprotein in an inactive state and prevents it from sending signals that drive uncontrolled cell growth. However, the targeted drug does not affect the wild-type KRAS protein (Lito et al., 2016; Patricelli et al., 2016).

#### 3.1.1 Sotorasib (AMG510)

A phase 1/2 clinical trial (NCT03600883, Table 2) showed promising results of sotorasib therapy among patients with advanced KRAS-G12C-positive solid tumors. According to the initial data shared in September 2020 (Hong et al., 2020), patients with NSCLC in the 960-mg cohort had an objective response rate (ORR) of 32.2% and a disease control rate (DCR) of 88.1%. Dose-limiting toxicity was not observed in safety studies, and treatment-related adverse events did not result in death in any case. The most common adverse reactions were diarrhea (29.5%), fatigue (23.3%), and nausea (20.9%). These results were recently validated by a phase II trial (Skoulidis et al., 2021), which included 122 patients with NSCLC. The ORR was 37.1%, with a median duration of response of 11 months and a DCR of 80.6%. The median progression-free survival (PFS) was 6.8 months, and the median overall survival (OS) was 12.5 months. In addition, a phase III clinical trial (NCT04303780, Table 2) is currently underway for comparing the efficacy of sotorasib and docetaxel in patients with NSCLC with KRAS G12 mutation before treatment. At present, sotorasib is undergoing phase II trials as a first-line treatment agent (NCT04933695, Table 2).

#### 3.1.2 Adagrasib (MRTX849)

Mirati Therapeutics developed MRTX849 (adagrasib), an orally bio-available covalent KRAS G12C inhibitor (KRAS G12C Inhibitor, Mirati Therapeutics, 2021). A phase 1/2 study (NCT03785249, Table 2) demonstrated the potential of MRTX849 as an effective and potent KRAS G12C inhibitor. The preclinical studies on adagrasib (Hallin et al., 2020) have revealed the following: ① Strong action: in multiple cell models of KRAS G12C mutation, adagrasib showed good efficacy at very low concentrations (nanomolar level). ② Long half-life: adagrasib is the only inhibitor of KRAS G12C mutant with a half-life of approximately 24 h. ③ High selectivity: the selectivity of KRAS G12C mutant was > 1000 times that of wild-type KRAS and other proteins. In a recent phase II single-arm trial, patients with advanced NSCLC who had previously received platinum-based dual-agent therapy and immune checkpoint inhibitor (ICI) therapy were enrolled. ORR was the primary endpoint as determined by an independent review board, whereas response duration, PFS, OS, and safety were secondary endpoints. As of 15 October 2021, 116 patients were enrolled. The median follow-up period was 12.9 months at the time of data analysis, and 98.3% of the patients had previously received chemotherapy and ICI therapy. There were 112 patients with evaluable lesions, with an ORR of 42.9%, a response duration of 8.5 months (95% CI, 6.2–13.8 months), and a median PFS of 6.5 months. As of 15 January 2022, the median follow-up period was 15.6 months, and the median OS was 12.6 months (95% CI, 9.2–19.2 months). The intracranial ORR was 33.3% in 33 previously treated patients with stable central nervous system (CNS) metastasis. Approximately 44.8% of treatment-related adverse events of ≥ grade 3 were observed, with the most common toxic effects being observed in the gastrointestinal (diarrhea, nausea, and vomiting) and hepatic (elevated liver enzyme levels) systems (Jänne et al., 2022).

### 3.2 Resistance mechanism and overcoming strategy of KRAS G12C-targeted drugs

Despite the availability of therapeutic agents for targeting KRAS G12C mutations and positive clinical outcomes, patients and physicians have to deal with the possibility of cancer cells developing resistance to sotorasib and adagrasib during clinical trials and actual treatment. Drug resistance mechanisms can be divided into two types: intrinsic and acquired drug resistance mechanisms.

#### 3.2.1 Low dependence on KRAS signaling

Intrinsic resistance refers to the presence of drugs before treatment. Some preclinical studies have shown that intrinsic resistance may be one of the important reasons for the heterogeneous response of patients to clinically targeted KRAS G12C therapy, which may result from the low dependence on KRAS signaling (Canon et al., 2019; Jiao and Yang 2020). As mentioned above, the RAS protein functions through various pathways, mainly including the mitogen-activated protein kinase (MAPK)–ERK and PI3K–AKT–mTORC1 pathways. Previous studies have shown that all KRAS-mutant cells do not depend on KRAS activation to maintain survival and may have different levels of dependence on KRAS signaling. In a study, activation of the two major downstream effectors, namely, ERK and AKT, was not inhibited after KRAS knockdown (Singh et al., 2009). Another study showed that some KRAS-independent cell lines or those with a low level of dependence survived even after KRAS was completely inhibited. In addition, a majority of these cells showed PI3K-dependent MAPK pathway activation, indicating that inhibition of the MAPK pathway was more extensive and effective (Muzumdar et al., 2017). These findings suggest that MAPK pathway inhibitors are potential options for overcoming KRAS G12C-targeted drug resistance.

AMG510 and MRTX849 have shown good therapeutic results in clinical trials. However, rapid adaptive resistance and MAPK signal reactivation after inhibitor treatment have been reported (Ryan et al., 2020). Complementary therapeutic strategies may help to realize the full potential of targeting KRAS mutants for cancer treatment. Proteolysis-targeting chimera (PROTAC)-mediated degradation serves as a complementary strategy for regulating KRAS mutants. PROTACs are emerging drugs that degrade target proteins through cellular proteasome degradation mechanisms. In 2020, Bond et al. (Bond et al., 2020) developed the PROTAC molecule LC-2 that degrades KRAS G12C mutants. LC-2 covalently binds KRAS G12C to an MRTX849 warhead, inducing rapid and continuous degradation of KRAS G12C, leading to the inhibition of the MAPK signal in KRAS G12C-mutant cell lines. It reduces oncogenic KRAS levels and downstream signal transduction in cancer cells and is the first reported compound that can degrade endogenous KRAS G12C.

#### 3.2.2 Synthesis of novel KRAS G12C protein

In a study (Xue, 2020), the main mechanism underlying the resistance of cancer cells to KRAS mutant inhibitors was investigated, and KRAS was found to function in both activated and inactivated states, whereas inhibitors were found to act only on inactivated KRAS. When inhibitors act on tumor cells, the function of KRAS is inhibited. However, some tumor cells can bypass this effect and resume proliferation. This rapid differential response occurs because some quiescent cells produce a new KRAS G12C protein in response to the inhibition of MAPK signaling. The newly synthesized KRAS G12C protein is maintained in an active, drug-insensitive state by epidermal growth factor receptor and Aurora kinase signaling. Cells that do not have these adaptations, or are inhibited by drugs, remain sensitive to drug treatment.

#### 3.2.3 RTK-mediated activation of wild-type RAS

RTKs are the largest class of enzyme-associated receptors, which are cell surface receptors for many growth factors, cytokines, and hormones. Studies have shown that RTK-mediated activation of wild-type RAS (NRAS or HRAS) can drive the inhibition of the KRAS pathway and drug resistance caused by KRAS G12C inhibitors (Jiao and Yang 2020; Ryan et al., 2020). Therefore, the use of RTK inhibitors may be a potentially effective strategy for overcoming the resistance to KRAS G12C inhibitors.

As discussed above, feedback reactivation mediated by multiple RTKs is a key mechanism for developing resistance to KRAS G12C-targeted agents. Targeting transduction molecules downstream of RTKs, such as SHP2 and SOS1, is one of the primary focus areas of current clinical research. SHP2 is a protein tyrosine phosphatase that activates the SOS-regulated RAS–GTP load to mediate cellular signaling *via* the RAS/MAP kinase signaling pathway (Ruess et al., 2018). Overactivation of SHP2 can stimulate the activation of several signaling pathways, including RAS–MAPK, Akt, and STAT5, thereby promoting the occurrence and development of tumors (Dong et al., 1996). Therefore, targeting SHP2 and SOS1 can be combined with KRAS G12C inhibitors. SHP2 is a phosphatase associated with the activation–inactivation mechanism of KRAS. In a study, a biopsy was performed on a patient with advanced lung cancer treated with AMG 510, and the potential drug resistance factors were identified. Because HER2 activation mediates resistance to targeted drugs in EGFR-mutated lung cancer, the study assessed its functional relevance in KRAS G12C-positive lung cancer. In controlled trials, sotorasib treatment combined with the SHP2 inhibitor TNO155 (NCT03114319, Table 2) has been found to synergistically inhibit cell proliferation and clonal growth in KRAS G12-positive models of HER2 overexpression (Ho et al., 2021).

#### 3.2.4 Mechanisms of acquired drug resistance

Acquired resistance mechanisms of KRAS G12C inhibitors can be divided into the following three categories (Awad et al., 2021): 1) On-target resistance of KRAS G12C inhibitors: KRAS G12C mutation transforms to G12D/R/V/W and other subtypes or G12C amplification. 2) Histological transformation: lung adenocarcinoma with KRAS G12C mutation can be histologically transformed to squamous cell carcinoma. 3) Off-target resistance of KRAS G12C inhibitors: acquired bypass resistance mechanisms include MET amplification; activation mutations of NRAS, BRAF, MAP2K1, and RET; oncogenic fusing of ALK, RET, BRAF, RAF1, and FGFR3; and loss-of-function mutations in NF1 and PTEN. Recent studies (Adachi et al., 2020) have shown that activation of epithelial–mesenchymal transition (EMT) leads to primary and acquired resistance to KRAS G12C inhibitors. In cells with KRAS G12C inhibitor resistance, EMT is induced *via* activation of the PI3K pathway, which eventually leads to the occurrence of drug resistance. In addition, AMG 510 increases PD-1 expression on CD8+ T cells, which may lead to immunosuppression of the tumor immune microenvironment, leading to secondary drug resistance.

Immune checkpoint antibodies block the PD-1 pathway by targeting PD-L1 or PD-1 and have shown good clinical efficacy in treating various malignancies, including NSCLC, in which KRAS/RAF mutations are the common driving events (Brahmer et al., 2015). A meta-analysis conducted by Kim et al. (2017) showed that compared with docetaxel, ICIs improved the overall survival rate of patients with advanced NSCLC with KRAS mutation. In a preclinical study on AMG 510 monotherapy, AMG 510 degraded tumors but did not cure them in the absence of immune cells in mice models, indicating that immune ability can promote the therapeutic effects of AMG 510 in mice with tumors. Subsequently, Amgen proposed the combination of AMG510 and the pembrolizumab antibody, and in 9 of 10 mice treated with this combination therapy, tumors disappeared permanently, significantly improving survival. In addition, mice treated with the combination therapy developed the ability to reject KRAS G12D tumors, suggesting that the combination therapy drives an acquired immune response. The combination of AMG 510 and anti-PD-1 antibody enhances tumor-specific T-cell response and further enhances antitumor T-cell activity. Amgen is currently testing this combination in patients with NSCLC (Canon et al., 2019). In a study, compared with monotherapy, the combination of AMG 510 and an MAPK inhibitor significantly enhanced the antitumor activity both *in vitro* and *in vivo* (Canon et al., 2019).

Therefore, combination therapy is necessary to improve the treatment of KRAS G12C-positive NSCLC and reduce the impact of drug resistance on the therapeutic efficacy. To date, many preclinical studies have provided a theoretical basis for combination therapy to enter clinical trials.

### 3.3 Prognostic nature of KRAS G12C mutation in non-small-cell lung cancer treated with different strategies

At present, the treatment of NSCLC mainly includes surgery, chemotherapy, immunotherapy, and targeted therapy. To improve therapeutic guidance in clinical practice, we examined the prognostic characteristics of the KRAS mutation subtypes in NSCLC treated with different regimes based on previous studies.

Some studies have shown that KRAS mutation is an independent prognostic factor for the poor prognosis of patients with NSCLC, which indicates shortened OS and an increased risk of tumor recurrence (Renaud et al., 2015). Compared with the KRAS wild-type, the KRAS G12C mutation suggests a poor prognosis. PFS and OS rates are lower in patients with KRAS G12C mutation receiving different treatment schemes than in patients with other KRAS mutation subtypes (Izar et al., 2014; Svaton et al., 2016; Park et al., 2017; Liu et al., 2020; Finn et al., 2021). If the therapeutic efficacy of ICIs (Wu et al., 2021) and the resection of stage I lung adenocarcinoma are favorable, better DFS is observed (Nadal et al., 2015). In a study involving 75 patients with clinical stage II–IV KRAS-mutant NSCLC, KRAS G12C mutation was identified as a predictive biomarker for better survival benefits from first-line chemotherapy in patients with advanced NSCLC with KRAS mutations (Lei et al., 2020). In another study on patients with advanced NSCLC who received EGFR–TKI treatment, KRAS G12C mutation was identified as a strong negative predictor of therapeutic benefits (Fiala et al., 2013). In addition, KRAS G12C mutation predicts worse PFS, whereas other KRAS mutants predicted better PFS in patients with NSCLC receiving targeted therapy (Ihle et al., 2012) (Table 1).

**TABLE 1:** Selected major studies about the prognostic relevance of KRAS G12C status in lung cancer.

Authors	Pts	Treatments	Comparison	Findings	Results
Liu. (2020)	(Stage I–IV) N = 130	Various	KRAS G12C vs. KRAS WT	1. OS: KRAS G12C vs. KRAS WT (18.3months vs. 26.7 months)	Poor prognosis
Finn et al. (2021)	(Stage I–III) N = 1014	Various	KRAS G12C vs. KRAS other	1. OS: KRAS G12C vs. KRAS other (HR = 1.39; 95% CI: 1.03–1.89, *p* = 0.031)	Poor prognosis
			KRAS G12C vs. KRAS WT	2. OS:KRAS G12C vs. KRAS WT (HR = 1.32; *p* = 0.028)	
Park et al. (2017)	(Stage IIB–IV) N = 334	Chemotherapy	KRAS G12C vs. KRAS WT	1. PFS:KRAS G12C vs. KRAS WT (HR = 1.96; *p* = 0.045)	Poor prognosis
Svaton et al. (2016)	(Advanced NSCLC) N = 127	Chemotherapy	KRAS G12C vs. KRAS other	1. OS: KRAS other vs. KRAS G12C mut (median 10.3 vs. 6.4 months, *p* = 0.011).	Poor prognosis
			KRAS mut vs. KRAS WT	2. OS: KRAS WT vs. KRAS mut (16.1 vs. 7.2 months, *p* = 0.008).	
Lei et al. (2020)	(Stage IIIB–IV) N = 75	Chemotherapy	KRAS G12C vs. KRAS other	1. PFS: KRAS G12C VS. KRAS other (median 4.7 vs. 2.5 months, *p* < 0.05).	Better prognosis
Nadal et al. (2015)	(Resected lung adenocarcinoma) N = 179	Surgical operation	KRAS G12C vs. KRAS other	1. DFS: KRAS WT vs. KRAS mut (*p* = 0.009).	Poor prognosis
			KRAS WT vs. KRAS mut	2. DFS:KRAS G12C vs. KRAS other (*p* < 0.001).	
Izar et al. (2014)	(Resected stage I lung adenocarcinoma) N = 312	Surgical operation	KRAS mut vs. KRAS WT	1. DFS: KRAS mut vs. KRAS WT (HR = 3.62,*p* < 0.0001).	Better prognosis
			KRAS G12C/G12V mut vs. KRAS other	1. DFS: G12C/G12V mut vs. KRAS other (*p* = 0.0271)	
Wu et al. (2021)	N = 246	Immune checkpoint inhibitor (ICI)	Kras G12C vs. KRAS other	1. PFS:KRAS G12C vs. KRAS other (median 4.8 VS. 2.1 m)	Better prognosis
Fiala et al. (2013)	(Advanced NSCLC) N = 448	Targeted therapy	KRAS G12C vs. KRAS other	1. PFS: KRAS G12C vs. KRAS other (4.3 week vs. 9.0 week).	Poor prognosis
				2. OS: KRAS G12C vs. KRAS other (9.3 week vs. 12.1 week).	
Ihle et al. (2012)	(Patients with refractory non-small-cell lung cancer) N = 341	Targeted therapy	KRAS G12C vs. KRAS other	1. MST (median survival time)	Poor prognosis
				KRAS G12C/G12V vs. KRAS other	
				1.84 VS. 3.35 months.	

KRAS WT, KRAS wild type; KRAS other, KRAS mutant subtypes that do not contain the KRAS G12C mutation.

## 4 KRAS G12V mutation in non-small-cell lung cancer

The KRAS G12V mutant has a valine residue (glycine at position 12 is mutated to valine). The KRAS G12V mutation occurs in approximately 21% of patients with NSCLC with KRAS mutations (Xie et al., 2021). Individuals who smoke are more predisposed to NSCLC with KRAS G12V mutation.

### 4.1 Prognostic and predictive role of KRAS G12V mutation in non-small-cell lung cancer

In a study by Jia et al. (2017) on Chinese patients with advanced NSCLC who received cisplatin- or carboplatin-based chemotherapy, PFS was significantly shorter in patients with advanced NSCLC with KRAS G12V mutation than in patients with wild-type KRAS or other KRAS mutations. Another study showed that KRAS mutation subtypes can predict disease recurrence and metastasis after surgical treatment of NSCLC, and patients with NSCLC with KRAS G12V mutation are predisposed to pleura–pericardial metastasis after surgery (Renaud et al., 2016). In addition, patients with KRAS G12V-positive lung cancer have a poor OS and a high recurrence rate.

### 4.2 KRAS G12V mutation treatment strategies

KRAS mutations may be a potential biomarker for predicting the survival benefit of immunotherapy. Lan et al. (2018) reported that the expression of PD-L1 is significantly correlated with KRAS gene mutations. Pan et al. (2021) reported that the expression of PD-L1 was higher in patients with NSCLC with KRAS G12V mutation than in patients with wild-type KRAS. KRAS G12V mutation can induce PD-L1 expression through the transforming growth factor-β/EMT signaling pathway to promote the immune escape of KRAS-mutant NSCLC. Therefore, the beneficial effects of PD-1/PD-L1 immunotherapy may be better among patients with NSCLC with KRAS G12V mutation than among patients with other KRAS mutations.

In a study on the clinical efficacy of chemotherapeutic agents in patients with advanced NSCLC with KRAS mutations, platinum-based chemotherapy with taxane had the best ORR, especially when used in combination with bevacizumab. Compared with pemetrexed, taxanes significantly improve PFS. The KRAS mutation subtypes respond differently to chemotherapeutic regimens. In particular, patients with KRAS G12V mutation respond well to taxane treatment (Mellema et al., 2015). In a recent retrospective study, patients with KRAS G12V- or G12A-positive advanced NSCLC who received first-line platinum-based chemotherapy with taxane had longer PFS than those who received platinum-based chemotherapy with pemetrexed or gemcitabine. In addition, compared with wild-type KRAS, KRAS G12V mutation is associated with increased sensitivity to cisplatin in NSCLC cell lines (Garassino et al., 2011).

## 5 KRAS G12D mutation in non-small-cell lung cancer

The KRAS G12D mutation refers to the presence of an aspartic acid residue (glycine at position 12 is mutated to aspartic acid). KRAS G12D mutations occur in approximately 15% of patients with NSCLC with KRAS mutations (Xie et al., 2021). Epidemiologically, KRAS G12D mutation is more likely to occur in patients with NSCLC who do not smoke.

The use of PD-L1 inhibitor therapy in patients with KRAS mutations remains controversial. A study on the influence of KRAS mutations on immune biomarkers revealed a significantly decreased expression of PD-L1 protein and immune cell infiltration (activated CD4 memory T cells, helper T cells, M1 macrophages, and NK cells) in the KRAS G12D/TP53 mutant group. KRAS G12D/TP53 co-mutation drives immune suppression and may serve as a negative predictive biomarker (Gao et al., 2020).

## 6 Discussion and outlook

KRAS mutations occur in a significant proportion of patients with NSCLC; therefore, early detection and treatment are critical. The genetic characteristics, mutational mechanisms, and susceptibility of various KRAS mutant subtypes to treatment should be extensively investigated. In terms of diagnostic technology, liquid biopsy has been continuously improved and optimized since the discovery of circulating tumor cells in the blood and the introduction of precision oncology. Liquid biopsy can allow early detection of cancer and is more sensitive than traditional cancer screening methods such as radiology or imaging. In addition, it can decrease overall healthcare costs (Heitzer et al., 2019). However, the development of new detection methods is bound to encounter biological and technical challenges. Recently, a study from the Italian Scientific Society discussed the most pressing technical issues in liquid biopsy with the hope of improving its application in clinical practice (Russo et al., 2021).

In terms of treatment, research into KRAS G12C-targeted drugs has offered novel therapeutic strategies for patients with KRAS mutations. However, targeted resistance will inevitably occur in clinical settings. Therefore, the identification of patients who might benefit from targeted monotherapy or combination therapy is important, and methods to identify such patients should be developed. In addition, the selection and implementation of a combination therapy regimen are important to improve the therapeutic efficacy and reduce the incidence of side effects. Furthermore, the treatment of KRAS-mutant cancer should not be limited to the currently available treatment strategies. In recent years, mRNA-based immunotherapy has entered clinical trials and achieved success in the treatment of solid malignancies. Moderna introduced G12C, G12D, G12V, and G13C mutant antigen candidate mRNA products in 2017, which entered phase I clinical trials in 2019 (NCT03948763, Table 2) (Bear et al., 2021; Miao et al., 2021). Future studies should be focused on examining the relationship between the immunological microbial environment and KRAS-induced immune responses. Other cancer therapies, including cutting-edge techniques such as RNAi and CRISPR technologies, which can be used to knockdown genes, transfer payloads into tumors, and activate the immune system, may also help to eliminate KRAS-mutated tumors. In addition, novel therapeutic strategies should be developed for the future treatment of cancer.

**TABLE 2:** Ongoing clinical trials related to KRAS mutated NSCLC.

Drug	Mechanism of action	Clinical trials	Phase	Clinical staging associated with NSCLC
AMG510	KRAS G12C inhibitor	NCT03600883	Ⅰ/Ⅱ	Advanced with KRAS G12C mutation
AMG510	KRAS G12C inhibitor	NCT04303780	Ⅲ	Advanced with KRAS G12C mutation
AMG510	KRAS G12C inhibitor	NCT04933695	Ⅱ	Advanced with KRAS G12C mutation
MRTX849	KRAS G12C inhibitor	NCT03785249	Ⅰ/Ⅱ	Advanced with KRAS G12C mutation
TNO155	SHP2 inhibitor	NCT03114319	Ⅰ	Advanced with KRAS G12C mutation
mRNA-5671	mRNA-derived KRAS targeted cancer vaccine	NCT03948763	Ⅰ	Advanced with KRASG12C, G12D, G12V, or G13C mutation
